# Qualitative shape from shading

**DOI:** 10.1177/20416695251338721

**Published:** 2025-05-30

**Authors:** J. Farley Norman, James T. Todd

**Affiliations:** 1 Western Kentucky University, Bowling Green, KY, USA; 2 7315 Ohio State University, Columbus, OH, USA

**Keywords:** three-dimensional perception, contours/surfaces, objects and features, shape

## Abstract

When human observers are asked to describe the shape of a surface, they often identify an arrangement of surface features like bumps, dimples, ridges, or valleys. The central hypothesis of the present research is that the perceptual representation of three-dimensional shape has a graph-like structure that is defined by patterns of surface curvature, and that this is the structure that artists depict when they produce line drawings of objects. Two experiments were performed, in which observers marked the boundaries of bumps on a shaded surface, or the locations of ridges and valleys. Although they were not specifically instructed about where those features were located, the observers’ responses corresponded quite closely with the curvature extrema on each depicted object, and their judgments exhibited a high degree of constancy over changes in the pattern of illumination. The relationship is much weaker between the perceived locations of ridges and valleys and the local extrema of luminance in an image. Although variations of luminance are strongly influenced by the pattern of surface curvature, they are also influenced by local variations in illumination caused by multiple light sources, cast shadows, or indirect reflections. Human observers can somehow distinguish between those two components of luminance variation, but the visual information that makes that possible has yet to be determined.

## How to cite this article

Norman, J.F. & Todd, J.T. (2025). Qualitative shape from shading, *i–Perception, 16*(0), 1–26. https://doi.org/10.1177/20416695251338721.

## Introduction

**Figure 1.**
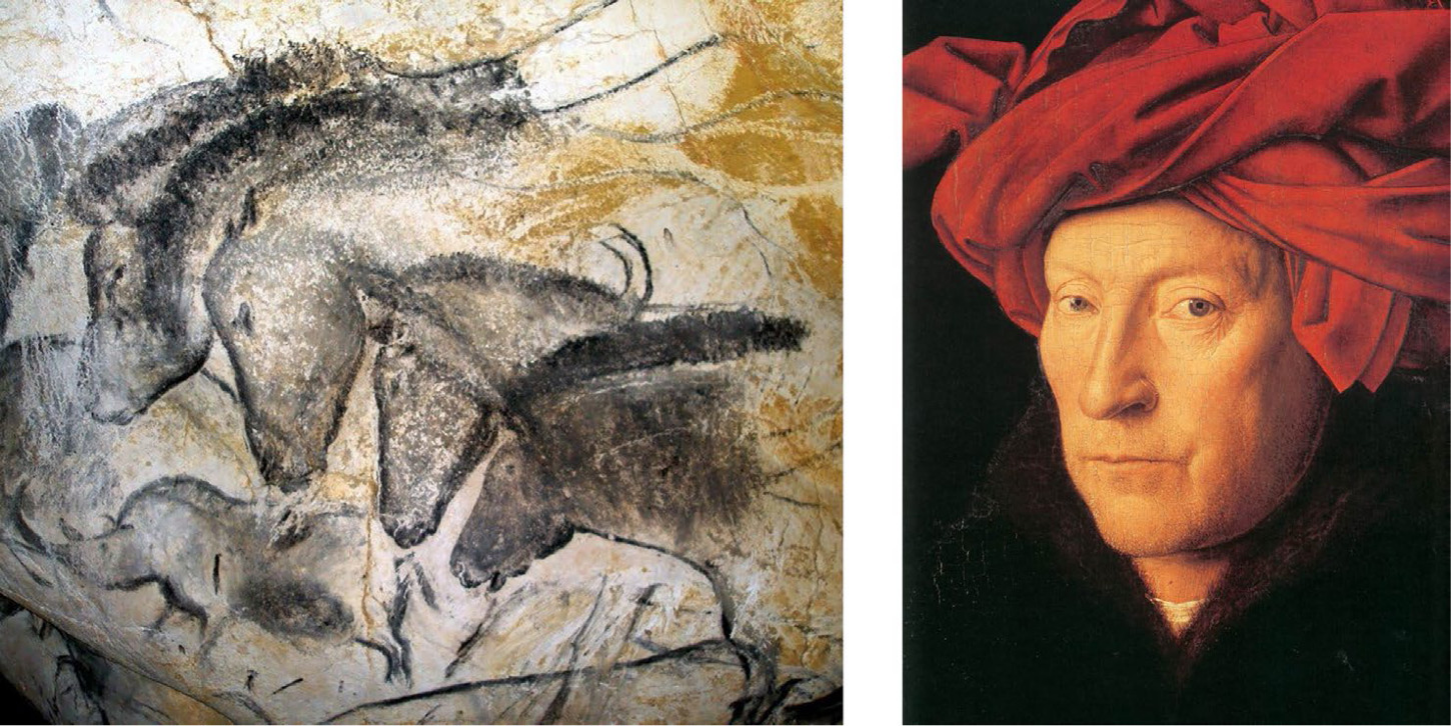
Examples of chiaroscuro from different periods of human history. Left: a painting of horses from the Chauvet cave in France, which was created around 30,000 BC. Right: a self-portrait of the Flemish Artist Jan van Eyck from 1433.

Patterns of image shading provide a powerful source of pictorial information for depicting the three-dimensional (3D) shapes of smoothly curved surfaces. In art, the manipulation of shading is often referred to as chiaroscuro. It is derived from the Italian word *chiaro* that means light, and the word *scuro* that means dark. The invention of this technique is often attributed to Leonardo da Vinci at the end of the fifteenth century, but it is actually much older than that. For example, the left panel of Figure 1 shows a painting of horses from the Chauvet cave in France, which was created around 30,000 BC. Note how the manipulation of shading produces a sense of volume in the figures. The right panel of Figure 1 shows what is believed to be a self-portrait of the Flemish artist Jan van Eyck from 1433. This is one of the earliest examples from modern western art that shows a true mastery of chiaroscuro to create a photorealistic depiction of skin and cloth.

How is it possible for variations of shading within a two-dimensional (2D) image to provide useful information about the 3D shapes of visible surfaces in the environment? The first computational models for estimating 3D shape from shading were developed by researchers in computer vision (e.g., Horn, 1975; Ikeuchi & Horn, 1981; Lee & Rosenfeld, 1985; Pentland, 1984, 1989). These models were designed to compute the depth or orientation of each local surface region based on some specific assumptions about what causes variations of image shading in the natural environment. One of these assumptions is that surfaces scatter light equally in all directions, which is often referred to as Lambertian reflectance. A second assumption is that the direction and magnitude of the incident light is the same over all visible surface regions, which is referred to as homogeneous illumination. Some models also require that the direction of illumination must be known.

In a general survey of shape from shading in computational vision, Zhang et al. (1999) performed simulations of 19 different algorithms that had been proposed in the literature at that time. The results of their analyses showed clearly that these algorithms produce systematic errors even when applied to synthetic images that satisfy their underlying assumptions. The results are even worse for photographs of objects or realistic renderings that do not satisfy the required assumptions, such as images that contain cast shadows or surface inter-reflections. What is particularly interesting is that many of these models produce erroneous features like ridges or valleys that are not physically present on the actual object.

**Figure 2.**
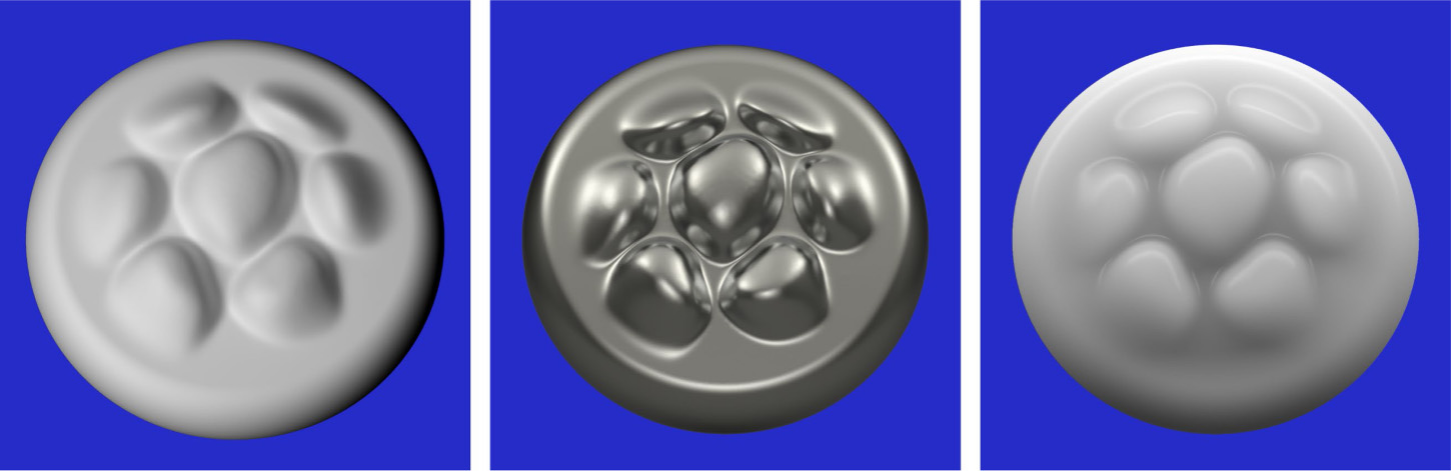
Three images of the same shape with different reflectance functions and patterns of illumination. Left: A surface with Lambertian reflectance illuminated by a single area light to the left. Middle: A polished metal surface that produces negligible scattering illuminated from multiple directions. Right: A translucent wax material illuminated from multiple directions. Some of the illumination is reflected from the surface with negligible scattering, and the remaining light is transmitted into the volume of the material where it is scattered uniformly in all directions.

One obvious problem for current models of 3D shape from shading is that the underlying assumptions on which they are based are almost never satisfied in the natural environment. Surfaces can reflect or transmit light in a wide variety of ways that bear little resemblance to Lambertian reflectance, and natural patterns of illumination contain numerous sources of inhomogeneity such as multiple light sources, cast shadows, or surface inter-reflections. Figure 2 shows three images of the same object with different material properties and patterns of illumination. Traditional models of shape from shading would make large systematic errors if applied to any of these images. For example, existing models would interpret the boundaries of the cast shadows as changes in surface orientation, and they would interpret specular highlights or reflections as bumps or dimples. Human observers, in contrast, are relatively unaffected by changes in surface reflectance or the pattern of illumination, such that all of the images in Figure 2 are perceived correctly as the same 3D shape.

Another potential problem with current computational models involves the specific representations of surfaces they are designed to compute. The output of these models is almost always a map of the local depths or orientations for all possible image locations. It is important to point out, however, that human observers are surprisingly imprecise when they are asked to make judgments about the magnitudes of depth or orientation differences between designated points on a smoothly curved surface (Norman & Todd, 1996; Todd & Norman, 1995). The Weber fractions for those judgments are typically many times larger than those obtained for other sensory properties, such as brightness, or the lengths and orientations of 2D lines. Moreover, when observers are asked to describe the shapes of surfaces like the ones in Figure 2, they never say anything about local depths and orientations. What they describe instead are qualitative surface features like bumps, dimples, ridges and valleys. For example, the object in Figure 2 is typically described as a rounded circular disk with seven small bumps.

In the present article, we will consider some alternative representations of 3D shape that are more closely aligned with observers’ descriptions. We will begin by examining how qualitative surface features like hills or valleys can be analyzed mathematically using differential geometry, and how the same analysis can also be used to describe patterns of shading within a visual image. Next, we will describe some novel psychophysical methods for measuring the perception of qualitative surface features, and we will present empirical evidence that observers can reliably identify those features in a manner that is largely invariant over changes in the pattern of illumination. Finally, we will also argue that the arrangements of these features have a graph-like structure that is exploited by artists to produce line drawings of objects.

### A Tutorial on Differential Geometry

**Figure 3.**
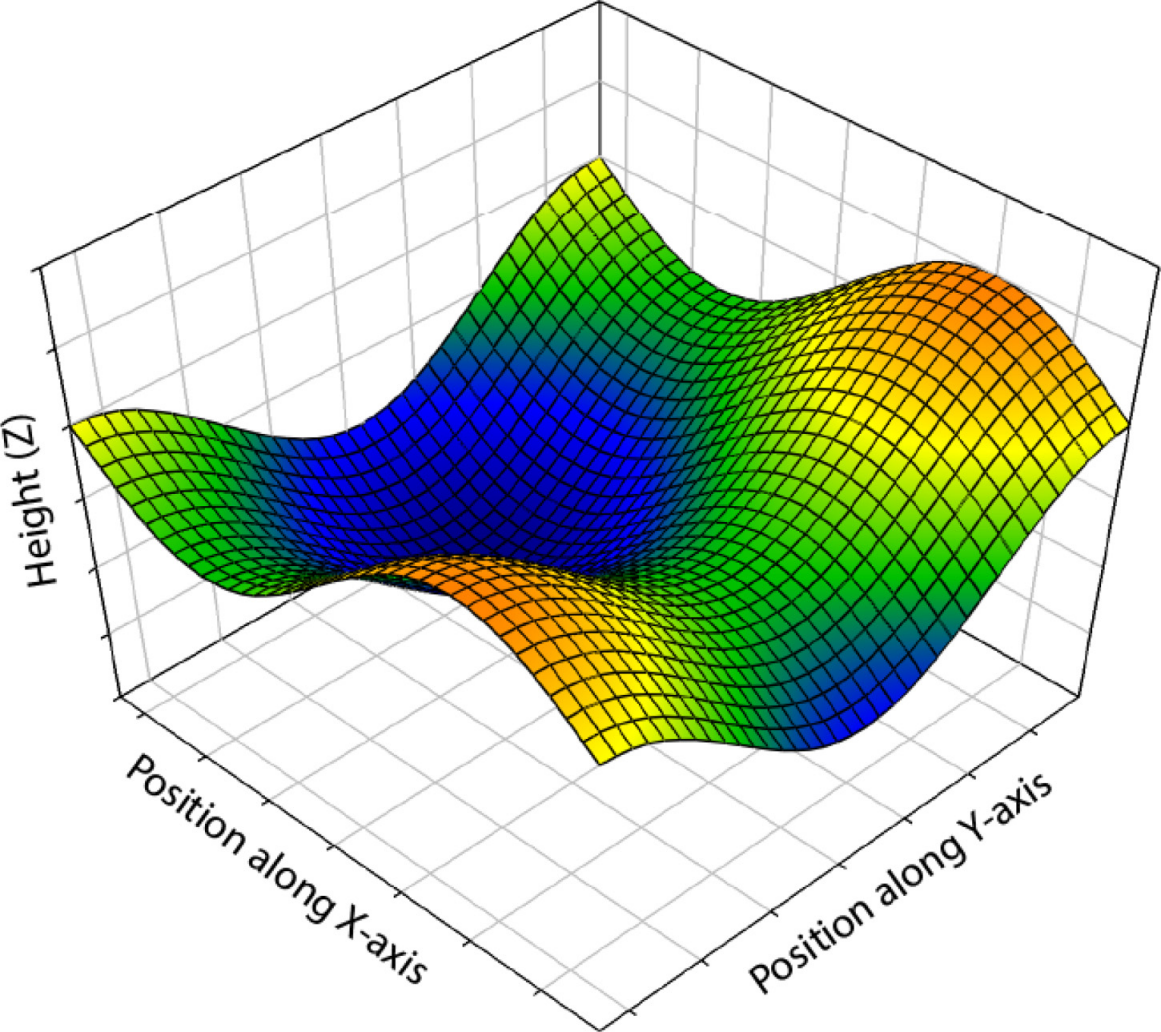
A mesh plot of a 3D surface that is positioned above a ground plane. At each point (*x*, *y*) on the ground plane, there is a corresponding height (*z*) on the depicted surface. These heights are color-coded in the image as a heat map to facilitate its perceptual interpretation.

To provide some background for the discussion that follows, it is useful to begin with a brief tutorial on differential geometry. Figure 3 shows a mesh plot of a 3D surface that is positioned above a ground plane. At each point (*x*, *y*) on the ground plane, there is a corresponding height (*z*) on the surface. Thus, the surface can be characterized as a scalar field that is defined by the following equation:

(1) z=f(x,y)

Within the context of visual perception, we can think about the *x*- and *y*-coordinates as visual directions, and (*z*) as the depth of the surface relative to the point of observation. The rate at which the depth (or height) changes between adjacent regions is what we intuitively recognize as the slope of a surface. This is defined by the first spatial derivatives of Equation 1: *f_x_* in the horizontal direction, and *f_y_* in the vertical direction. The local depth gradient (∇*f*) is a vector in the direction of maximum slope that is defined by:

(2) $$\nabla f=(\;f_{x},f_{y})$$

What we intuitively recognize as the local orientation of a surface at any given point can be defined in two ways. One is to describe the orientation of a plane that is tangent to the surface at that point. Another more common approach is to describe a vector with a length of one that is perpendicular to the surface. This is called a unit surface normal (*N*), and it is defined by:

(3) $$N=\frac{\left(\;f_{x},\;\;\;f_{y,}\;-1\right)}{\sqrt{\;f_{x}^{2}\;+f_{y}^{2}\;+1}}$$

**Figure 4.**
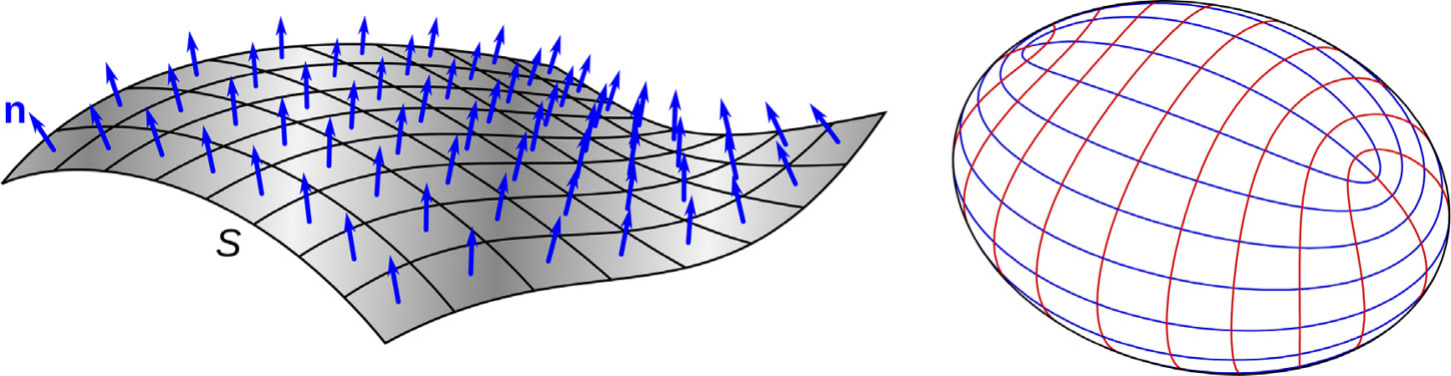
Left: A surface mesh composed of quadrilateral facets. The normal vector at the center of each facet is represented by a blue arrow. This image was created by Chetvomo and is licensed under cc-sa-1.0. Right: The lines of principal curvature on an ellipsoid. The lines of maximum curvature (κ_max_) are colored red, and the lines of minimum curvature (κ_min_) are colored blue. This image was created by Ag2gaeh and is licensed under cc-sa-4.0.

The left panel of Figure 4 shows a surface mesh composed of quadrilateral facets, and the normal vector at the center of each facet is represented by a blue arrow. Note that the orientations of these vectors change systematically across different regions of the surface. The rate of that orientation change is what we intuitively recognize as curvature, and it is defined mathematically by the second derivatives of Equation 1 (*f_xx_*, *f_xy_* and *f_yy_*). For most surfaces (excluding planes and spheres) there is one direction at each point where the curvature (κ_max_) is larger than any other, and another direction where the curvature (κ_min_) is smaller than any other. These are referred to as the principal directions of curvature and they are always orthogonal to one another. The right panel of Figure 4 shows the lines of principal curvature on an ellipsoid. These are curves that are aligned with a principal direction at each point through which they pass. The lines of maximum curvature (κ_max_) are colored red, and the lines of minimum curvature (κ_min_) are colored blue.

There are several other measures of curvature that are derived from the values of κ_max_ and κ_min_. One of these called Gaussian curvature is the product of those two components. It can be used to categorize surface patches into three general classes. For saddle-shaped surface regions, the two principal curvatures have opposite signs (i.e., one is concave, and one is convex) so that their product is negative. These are referred to as hyperbolic regions. For ridges or valleys, one of the principal curvatures is zero, so that their product is also zero. These are referred to as parabolic regions. Finally, for bumps or dimples, both principal curvatures have the same sign so that their product is positive. These are referred to as elliptic regions. One problem with Gaussian curvature as a measure of qualitative shape is that it cannot distinguish between bumps and dimples, or ridges and valleys.

Another classical measure called mean curvature is defined as the average of κ_max_ and κ_min_. It can distinguish surfaces that are uniformly concave from those that are uniformly convex, and it captures the overall magnitude of curvature for those surfaces. However, it can systematically underestimate the overall curvature of saddle-shaped surfaces because the positive and negative components cancel one another. Although other possible curvature measures have been proposed that have different strengths and weaknesses (Koenderink, 1990), the analyses described in the present article will rely primarily on mean curvature (see Appendix for a more detailed discussion of this issue).

We have focused our discussion thus far on the differential geometry of surfaces, but it is important to keep in mind that the same analysis is also applicable to images. The variable (z) in Equation 1 can be used to describe any possible scalar property that varies as a function of image position (*x*, *y*). This could be the depth (or height) of each visible surface region, but the same equation could also represent the luminance intensity of each visible surface region. Thus, all the tools of differential geometry can be brought to bear for the analysis of shaded images, including the computation of luminance gradients or luminance curvature. When considered from this perspective, the patterns of depths or luminance are both scalar fields with the same underlying mathematical form described by Equation 1.

### Singular Structures Within Scalar Fields

**Figure 5.**
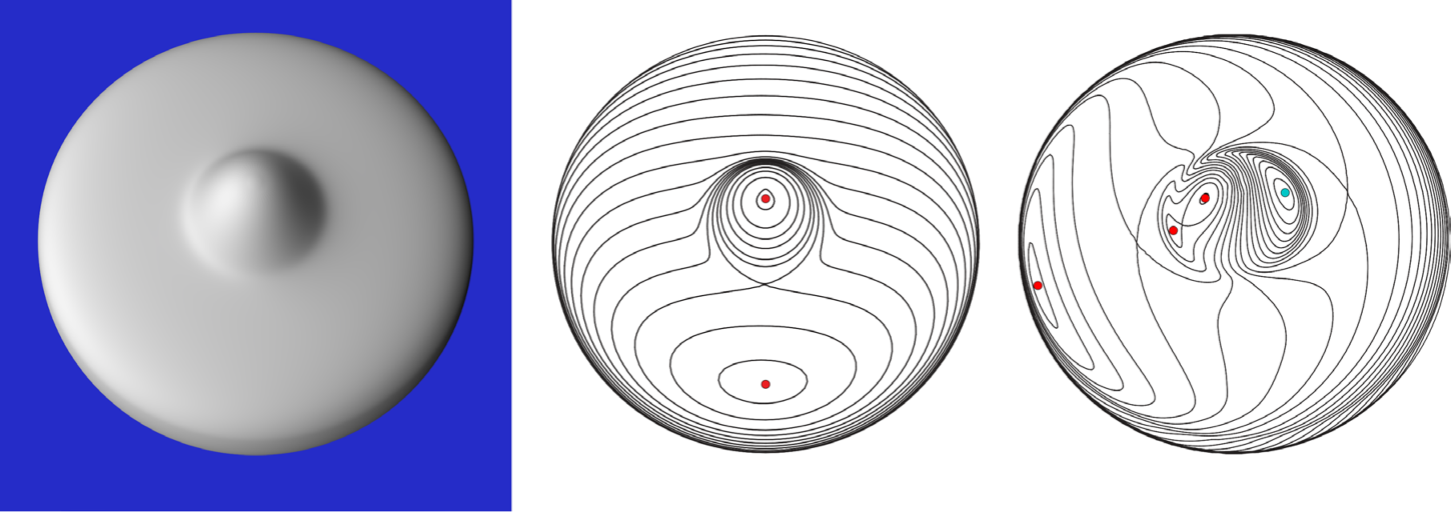
Left: An image of a rounded disk with a single bump at its center. Middle: The iso-contours of the surface depth field. Right: The iso-contours of the image luminance field. The red dots mark local maxima, and the cyan dots mark local minima.

One popular method for representing the structure of scalar fields is to plot a pattern of contours that connect neighboring points in (*x*, *y*) that have the same scalar value (*z*). These are sometimes referred to as iso-contours or level sets. The left panel of Figure 5 shows a rounded circular disk with a single bump at its center. This object has a Lambertian reflectance function, and it is illuminated by a large area light from the left. The middle panel shows the pattern of iso-depth contours for this object, and the right panel shows the pattern of iso-luminance contours for the shaded image. Note that these patterns are quite different from one another, which highlights the difficulties of computing shape from shading.

The small dots in Figure 5 mark singular points in the scalar fields where *f_x_* = *f_y_* = 0. Local maxima and minima are revealed in these plots where the contours become progressively smaller converging toward a single point. The red circles mark local maxima where both principal curvatures are positive, and the cyan circles mark local minima where both principal curvatures are negative. Saddle points are located in regions where the iso-contours cross one another. These are points where *f_x_* and *f_y_* are both zero, and the two principal curvatures have opposite signs. There are some strong topological constraints on the arrangements of these singularities, and a good readable discussion of that can be found in Kunsberg and Zucker (2021). For example, one such constraint that is easy to comprehend at an intuitive level is that two local maxima must always be separated by a local minimum or a saddle point.

### Visual Information About Qualitative Surface Structure

Let us now consider some possible sources of visual information from which the qualitative structure of surfaces could be perceptually specified. An early investigation of this problem was performed by Koenderink and van Doorn (1980) for the special case of Lambertian surfaces with homogeneous illumination from a distant point light source. They provided mathematical proof that saddle points in the luminance field under those conditions will always correspond to parabolic points on the physical surface. Kunsberg and Zucker (2018, 2021) have more recently noted that local luminance minima under homogeneous illumination are also generally associated with parabolic points on a surface. They developed an algorithm that exploits those features to construct a set of closed curves called critical contours that correspond to the boundaries of bumps. Each critical contour is anchored by a local minimum and a saddle point of the luminance field, and it connects points in an image where the luminance changes most rapidly along gradient flow lines perpendicular to the contour. For surfaces with Lambertian reflectance and homogeneous illumination, the critical contours will be approximately aligned with parabolic lines on the depicted surface. Parabolic lines are ideal candidates for defining the qualitative structure of smooth surfaces because they form the boundaries between regions with positive and negative Gaussian curvature.

Another possible strategy for defining the boundaries of surface features was suggested by Hoffman and Richards (1984). They were among the first researchers to argue that decomposing shapes into parts is a basic function of human perception, and they proposed that negative minima of curvature are ideal features to define part boundaries. One obvious problem for their proposal in the context of shape from shading is that they did not suggest any possible sources of information by which negative curvature extrema might be detected within the structure of the luminance field.

**Figure 6.**
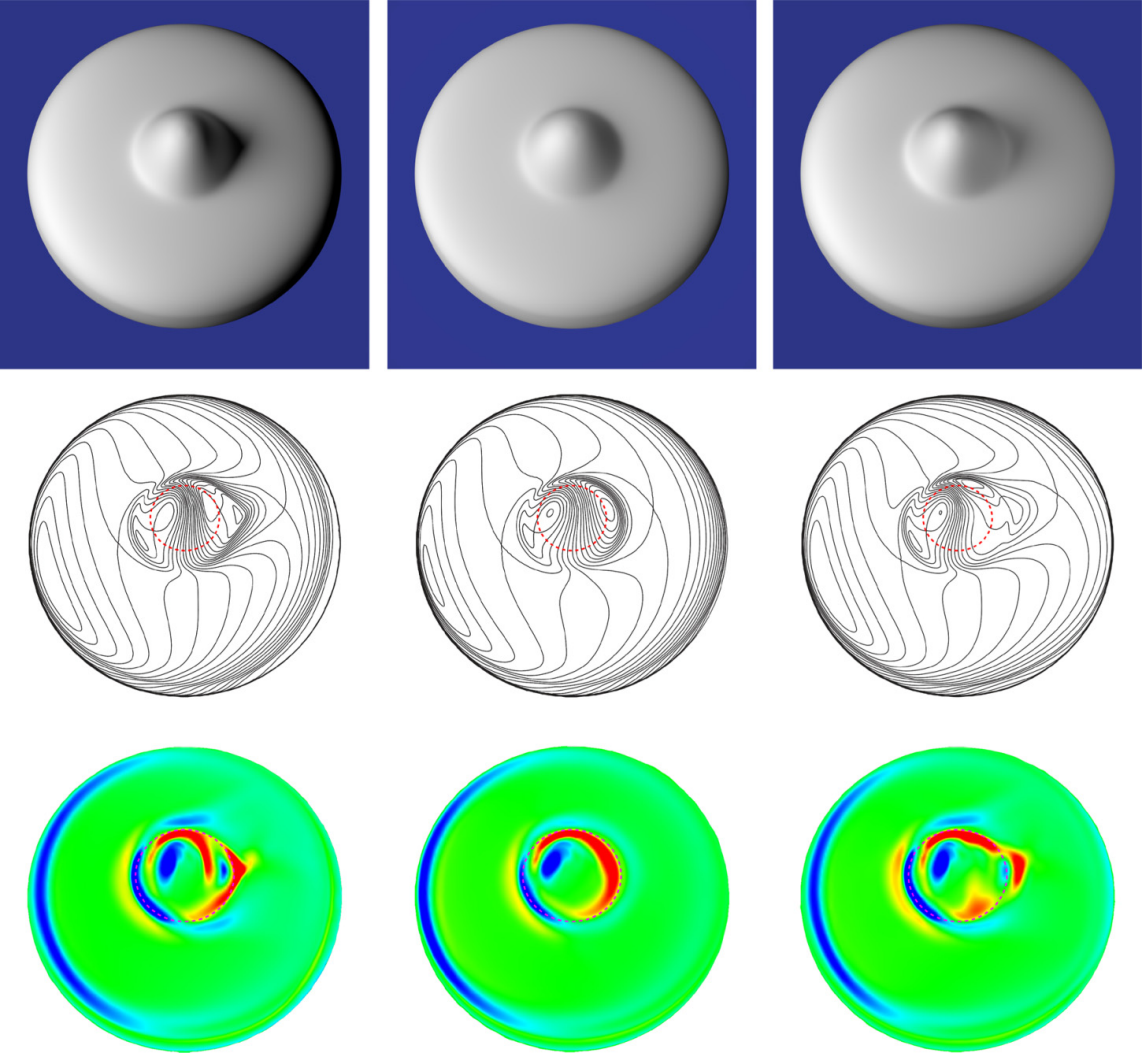
Top row: Three images of a rounded disk with a single bump at its center. All three objects have Lambertian reflectance functions with different patterns of illumination. Middle row: The iso-luminance contours for the images in the top row. The dotted red contours show the parabolic lines on each of the depicted objects. Bottom row: The luminance mean curvature for all of the images in the top row. The blue and cyan regions mark regions where the mean curvature of the luminance field is strongly positive, and the red and yellow regions mark regions where the mean curvature is strongly negative. The dashed magenta contour in each image shows the locus of points on the depicted object where the curvature in the principal direction is a negative extremum.

The top row of Figure 6 shows three different images of the same object. All three objects have Lambertian reflectance functions, but they have different patterns of illumination. The one in the left panel is illuminated by a small light to the left. This produces homogeneous illumination on the left side of the depicted bump, but most of the direct light is occluded from the right side, which produces a dark cast shadow. Note, however, that there is a relatively bright spot in the center of the shadow that is caused by indirect illumination from the base of the object. The object in the middle panel is illuminated by a much larger area light. Some of this direct light is able to reach the right side of the depicted bump, which softens the cast shadow. Another popular photographic technique for softening shadows is shown in the right panel. The object in that image is illuminated by a small bright “key” light on the left and a dimmer “frame” light on the right.

The middle row of Figure 6 shows the iso-luminance contours for all three of these images, together with dotted red contours that show the parabolic lines on the depicted objects. Note that there is a saddle point in the luminance field on the left side of the depicted bump for all three images. When the objects are illuminated by small lights that produce homogeneous illumination on the left side of the bump, these saddle points correspond to parabolic points on the depicted surface as predicted by Koenderink and van Doorn (1980). However, for the object illuminated by a large area light in the middle panel, the saddle point is shifted slightly away from the surface parabolic line. The pattern of luminance within the shadows on the right of the bumps is more complex. For the surface in the middle panel, there is a single local minimum that is close to the parabolic line. That image is ideally suited for the analysis of Kunsberg and Zucker (2018, 2021), because the bump is defined optically by a single saddle point on the left and a single luminance minimum on the right. However, the other two images are more complex because of the cast shadows and multiple directions of illumination. In both of those luminance fields there are two local minima that are separated by an additional saddle point. It is not clear how Kunsberg and Zucker's model might deal with that additional complexity. The main thing to keep in mind from these examples is that the number of singularities in the luminance field can vary significantly over changes in the pattern of illumination, which alters its differential topology.

Another way of representing the structure of the luminance field is to create a map of the luminance curvature. The luminance gradient is the rate at which luminance changes at any given location in an image. Luminance curvature, in contrast, describes the rate at which the gradient changes. If you think about magnitudes of luminance as the height of a surface, then bright regions correspond to bumps or ridges with positive curvature, and dark regions correspond to dimples and valleys with negative curvature. Moreover, local extrema of luminance curvature in any given direction correspond to local extrema of luminance in that direction. The bottom row of Figure 6 shows the mean curvature of the luminance field for all of the images in the top row (see Appendix for a more detailed discussion of how these plots were created). Regions where the mean curvature is strongly positive are colored blue or cyan, and those where the mean curvature is strongly negative are colored red or yellow. The dashed magenta contour in each image shows the locus of points on the depicted object where the curvature in the principal direction is a negative extremum. (These contours should not be confused with the parabolic lines depicted in the middle row.) Note how the dashed contour passes through the center of the positively curved regions of the luminance field on the left, and that it hugs the boundaries of the negatively curved regions on the right. This could provide a potential source of visual information to support the hypothesis of Hoffman and Richards (1984) when applied to the analysis of 3D shape from shading.

Despite the elegant theoretical analyses of qualitative shape from shading that have been proposed in the literature (e.g., Han & Zickler, 2024; Koenderink & van Doorn, 1980; Kunsberg & Zucker, 2018, 2021), there has been very little research on the perceived qualitative structure of smoothly curved surfaces from patterns of image shading. Todd, Yu and Phillips (2023) showed that observers can identify surface concavities along designated scan lines of an image. Experiment 1 used a similar technique to measure the perceived boundaries of bumps. Are these boundaries defined by surface parabolic lines as suggested by Kunsberg and Zucker (2018, 2021) or are they defined by negative curvature extrema as argued by Hoffman and Richards (1984)? To address that question, observers were shown the three shaded images shown in Figure 6, and they were asked to mark the boundaries between the depicted bump and the rest of the object by positioning small dots along designated scan lines through each image.

## Experiment 1

### Methods

#### Stimuli

The stimuli consisted of three images of a rounded circular disk with a single bump in the center, which are shown in the top row of Figure 6. All of the depicted surfaces had Lambertian reflectance functions, but they had different patterns of illumination. The one in the left panel was illuminated by a small area light above and to the left of the object. The one in the middle panel was illuminated by a light that was 16 times larger at the same location. The one in the right panel was illuminated by the same small light as in the left panel, but a second dimmer light was also located to the right of the object. The images were created using the Maxwell renderer by Next Limit, with a spatial resolution of 1200 × 1200 pixels. The rendered images were globally tone mapped for the Apple monitor into the sRGB 2.1 color space with a D65 white-point and a gamma of 2.2. No other global histogram adjustments (e.g., tint or burn) or local sharpening or contrast enhancement operators were used. The images were presented within a 27.8 × 27.8 cm region of the display screen, and they were viewed from a distance of 57.3 cm. At that distance, 1 cm equals 1 degree of visual angle.

#### Procedure

On each trial, observers were presented with a single image, and a single dot was positioned at a random location along a radial line that was anchored at the optical center of the depicted bump. They were instructed to position the point at the boundary that separated the bump from the rest of the object. This was achieved by pressing four possible response keys, two that moved the dot forward and backward at a rapid rate to quickly get it in the approximate neighborhood of the boundary, and two that moved the dot at a slower rate to position it as accurately as possible. When they were satisfied with their response, a new trial was initiated by pressing the space bar on the computer keyboard. There were eight different scan lines for each image at 45° intervals around the optical center of the depicted bump. Thus, each observer marked eight points along the perceived bump boundary for each of the three possible images. One of the authors (JFN) and five naïve observers (3 males and 2 females) participated in the experiment, and an entire experimental session took about 45 min.

Note that the adjustments of the eight different scan lines were completely independent of one another, and that the adjusted positions were removed from view at the end of each trial. There were no constraints on the overall pattern of adjustments. For example, the adjusted dots could form a circular, elliptical, or star-shaped pattern if that is how the boundary was perceived. Similarly, the observers were given no instructions about where the boundary should be located (e.g., at parabolic points or extrema of negative curvature).

### Results

**Figure 7.**
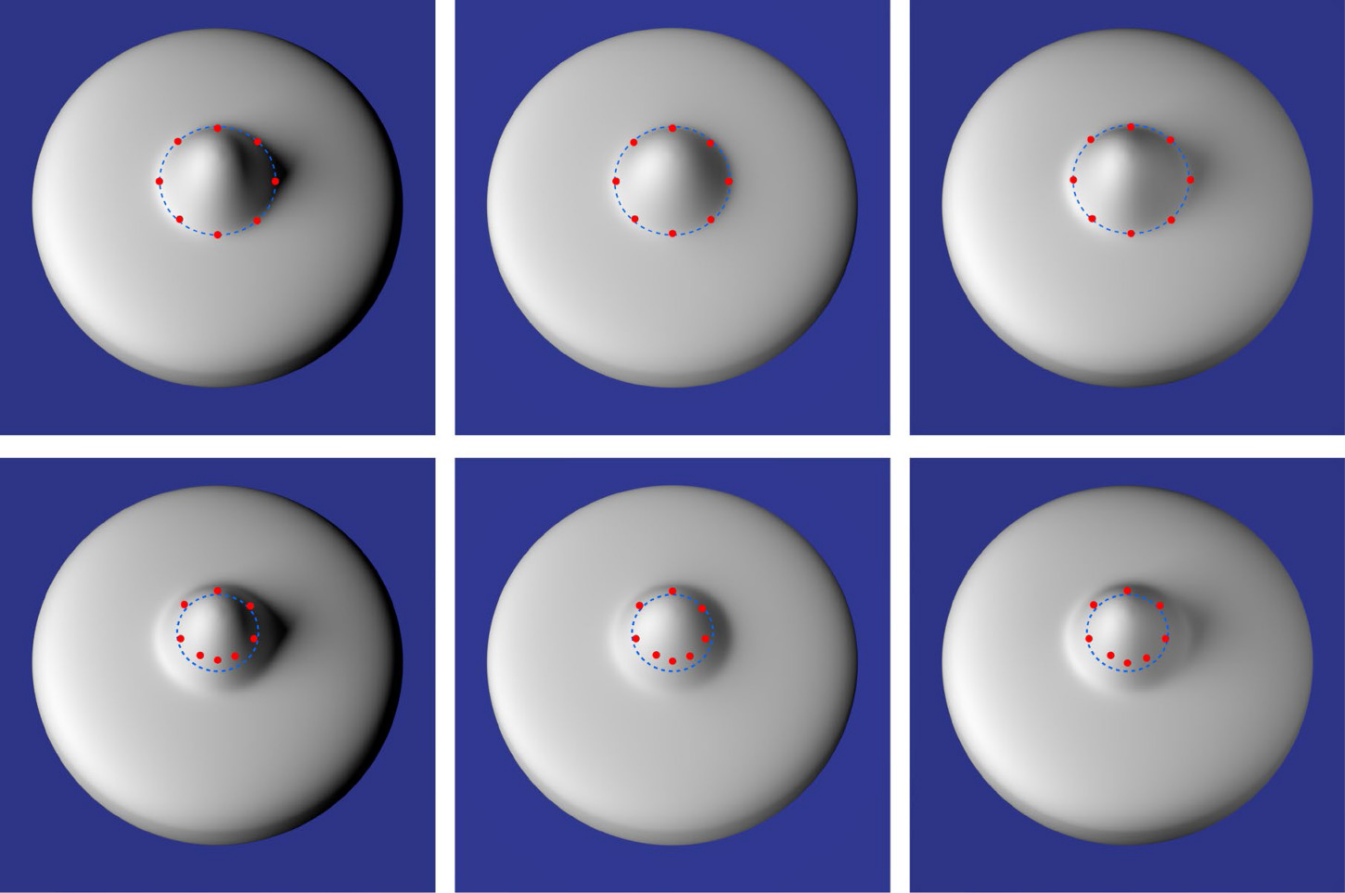
Results from Experiment 1. Top row: The red dots show the average judged boundaries of six observers between the bump and the rest of the object along the eight possible scan lines for each of the three stimulus images. The dashed blue contours show the lines on the depicted objects where the curvature in the principal direction is a negative extremum. Bottom row: In this case, the red dots show the average judged parabolic points of a single observer (over six sessions) between the regions with positive and negative curvature along the eight possible scan lines for each of the three stimulus images. The dashed blue contours show the parabolic lines on the depicted objects.

The top row of Figure 7 shows the overall pattern of results. The mean responses of all six observers along each scan line are marked by red dots that are superimposed on the stimulus images, and the dashed blue line shows the locus of points on the depicted object where the curvature in the principal direction is a negative extremum. There are several aspects of these results that deserve to be highlighted: First, the six observers were remarkably consistent with one another. The average between-subject standard error along each scan line was only 2.0 pixels (i.e., 2.8 min of visual angle). To put this in some perspective, the small red circles used to denote the responses in Figure 7 are 20 pixels in diameter. Thus, we did not include error bars in that figure because they would be too small to be visible.

A second thing to note in these data is that the observers’ judgments were almost perfectly aligned with the negative curvature extrema on the depicted object, and that they exhibited almost perfect constancy over changes in the pattern of illumination. This is quite obvious from the pattern of results depicted in Figure 7. However, to provide a more quantitative measure we computed the distance between the judged boundary for each scan line and the nearest local curvature extremum. Over the entire distribution of 24 scan lines, the average unsigned distance was just 3.1 pixels (i.e., 4.5 min of visual angle) and the standard error of that measure was only 0.46 pixels (i.e., 0.66 min of visual angle). It is important to keep in mind that the red circles in Figure 7 all have a diameter of 20 pixels, which provides a frame of reference to evaluate the precision and accuracy of the observers’ responses. It should also be noted that we provided no instructions to the observers about where the bump boundaries were located. They were free to define the boundary in any way that seemed intuitively plausible, yet their responses were all clustered tightly near the negative curvature extrema. This provides strong support for the part boundary hypothesis of Hoffman and Richards (1984).

The results of Experiment 1 show clearly that the bump boundaries of these stimuli are readily identified by naïve observers, and that they correspond quite closely to local negative extrema of surface curvature. Of course, this does not preclude the possibility that observers may also have knowledge about the boundary between regions with positive and negative curvature as described by Kunsberg and Zucker (2018, 2021). This could easily be tested by simply changing the instructions of the task, but our experience suggests that naïve observers with no knowledge of differential geometry would be unable to understand those instructions. We decided instead to test a single sophisticated observer (JFN) to see if he could identify the surface parabolic lines on the depicted surfaces. The procedure was the same as the one described above, except that the observer was specifically focused on marking the boundary that separates regions with positive and negative curvature. He performed that task for all three objects on 6 successive days.

The observer reported that this task is much more difficult than the previous one. Although he could clearly perceive that the top of the bump had a positive curvature and the bottom of the bump had a negative curvature, he had no clear sense of a specific boundary that separated those two regions. The results of his adjustments are presented in the bottom row of Figure 7. The mean responses over all six sessions are marked by red dots that are superimposed on the stimulus images, and the dashed blue contours show the parabolic lines on the depicted surfaces. The average standard error of those judgments was 2.1 pixels, which is comparable to the between-subject variance of the boundary identification task. However, the responses were systematically shifted relative to the parabolic line with a mean displacement of 16.3 pixels. Thus, these findings indicate that knowledgeable observers can make reliable judgments about surface parabolic lines when pressed to do so, but these judgments are systematically distorted relative to the ground truth.

A specific source of information that has been proposed in the literature for specifying parabolic points on a surface includes saddle points in the luminance field (Koenderink & van Doorn, 1980; Kunsberg and Zucker, 2018, 2021), but those points are often located in regions where spatial changes in luminance are quite small making them difficult to detect. Our results indicate that observers are more sensitive to extrema of surface curvature. Although luminance changes more rapidly in those regions, that is not by itself sufficient for the identification of curvature extrema. One possible source of information is the pattern of curvature in the luminance field. Note in the bottom row of Figure 6 that the curvature extremal contours on the depicted surface pass through positive extrema of luminance curvature on the side of a bump that faces the light source, and negative extrema of luminance curvature on the side that faces away from the light source. It is also important to note, however, that luminance curvature is also affected by local variations of illumination in addition to variations in the depicted surface curvature. Observers seem to be able to tease apart those separate sources of luminance variation, but it is not at all clear how that is possible.

## Experiment 2

The stimuli employed in Experiment 1 depicted a single bump on an otherwise convex surface. This is the simplest possible object that contains qualitative changes in the sign of surface curvature. In Experiment 2, we employed a different methodological procedure that makes it possible to investigate the perceived qualitative structure of more complex surfaces. Observers were shown shaded images of randomly shaped objects that contained multiple ridges and valleys in different orientations, and they were asked to mark central axes of those features by drawing over the images with a hand-held mouse.

### Methods

**Figure 8.**
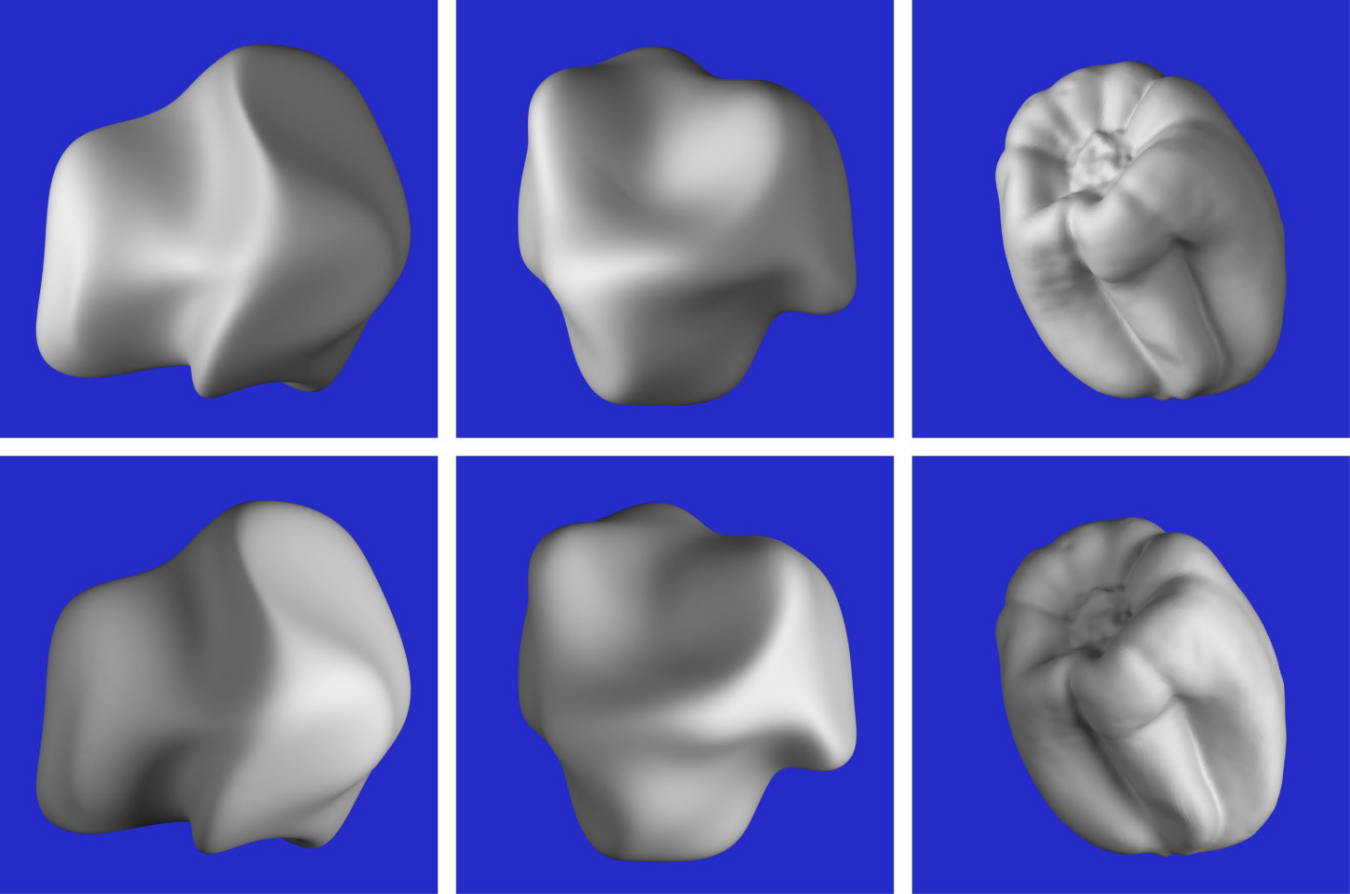
The six stimuli used in Experiment 2. The objects in the top row are illuminated by a bright light on the left and a dimmer light on the right. The ones in the bottom row are illuminated by a bright light on the right and a dimmer light on the left.

The stimuli consisted of six images that are shown in Figure 8. They depicted three different objects with two possible patterns of illumination. Two of the depicted objects were randomly deformed spheres (see Norman & Todd, 1996; Todd & Norman, 1995), and the other was a bell pepper that was digitized with a 3D scanner. Both patterns of illumination were produced by two small area lights, one that was located to the left of the object and another that was located on the right. One of these lights was always five times brighter than the other. The bright light was located on the left for the objects depicted in the top row of Figure 8, and it was located on the right for those in the bottom row. Note that these variations in illumination cause large differences between the structures of the luminance fields in the top and bottom rows of the figure. The average correlation between each corresponding pair of images (excluding the background) was −0.43.

The viewing conditions were identical to those described for Experiment 1. The task was performed using the Photoshop program by Adobe. Observers were presented with one of the six stimulus images, and they were asked to mark the ridges and valleys on the depicted object using the pencil tool. The ridges were marked on one layer and the valleys were marked on another, and the observers were allowed to switch back and forth. If they were unhappy with the placement of any given contour, they were allowed to delete it and replace it with another one. Once they were satisfied with their responses, they terminated that trial, and another new image would be presented. One of the authors (JFN) and six naïve observers (4 males and 2 females) participated in the experiment, and an entire experimental session took about 1.5 h.

### Results

Before describing the results, it is useful to consider the statistical degrees of freedom for this task. Each image contained 1.4 million pixels, roughly half of which were part of the blue background, and the remaining half provided information about the depicted object. Observers were required to select a subset of those pixels that best represented the ridges and valleys on the depicted object, and the number of marked pixels generally ranged between 3 and 6 thousand. Thus, observers’ judgments were highly selective in that they only marked about 1% of the visible surface regions. The key issues that need to be evaluated are the consistency of these judgments both within and between observers, and the extent to which they correspond to properties of the depicted surface and/or the luminance field.

**Figure 9.**
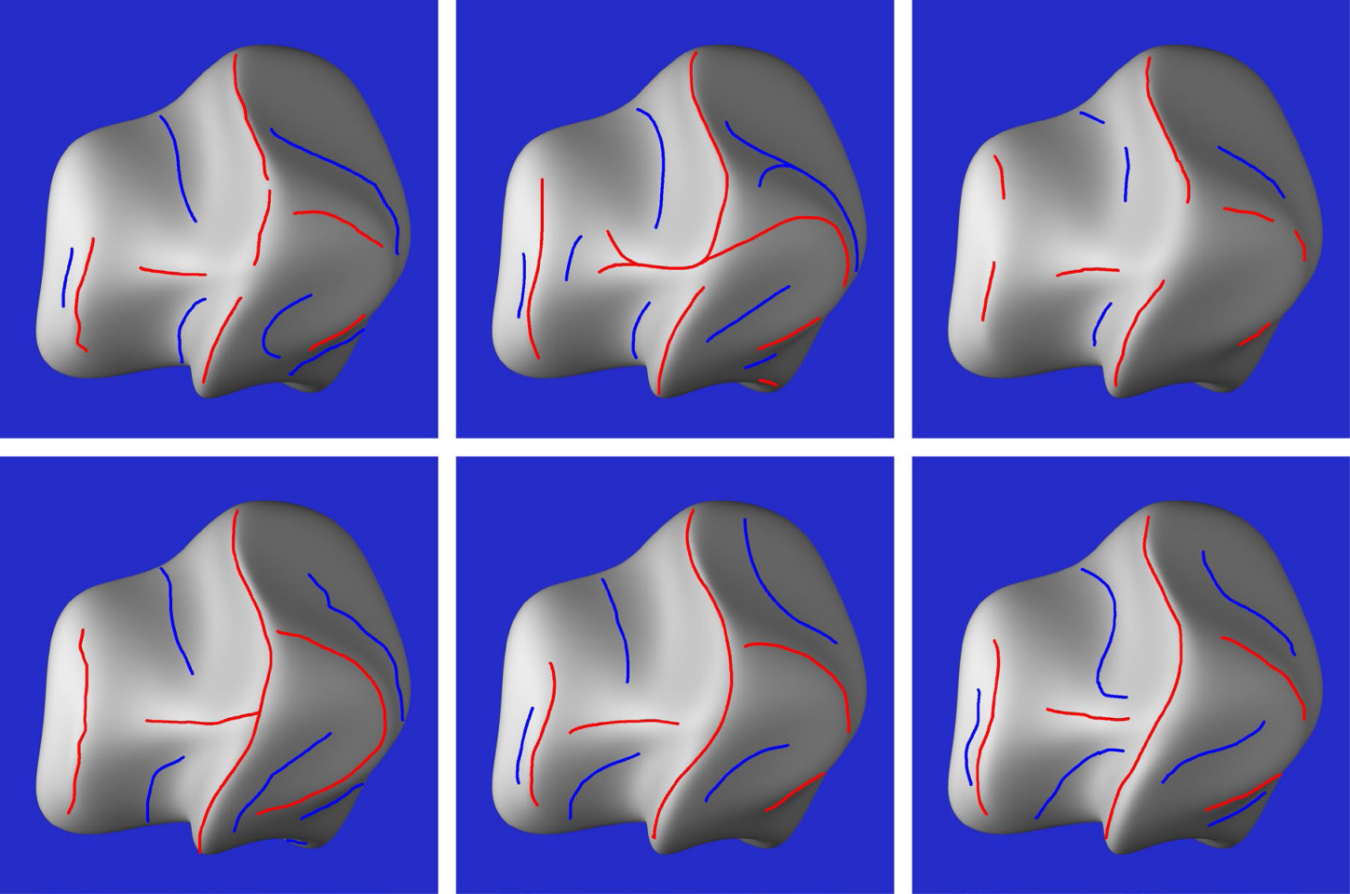
The responses of six different observers for one of the six stimulus images. The judged ridges are marked by red lines and the judged valleys are marked by blue lines.

Figure 9 shows the raw results from six observers for one of the six stimuli. Note that some observers had a higher threshold than others for selecting ridges and valleys, but the overall pattern of responses for all observers was quite similar. However, there were some clear individual differences for this particular stimulus. Consider the valley to the lower left of the prominent ridge in the center. For the response patterns in the top row, the observers marked the location of that valley along a bright region of the image (i.e., a luminance ridge). For the responses in the bottom row, the location of that valley was marked along a dark region of the image (i.e., a luminance valley). Similar individual differences also occurred for the valley to the upper left of the prominent ridge. Some observers marked it along a luminance ridge while others marked it along a luminance valley.

To facilitate a formal analysis of these data, we created a new image for each observer, in which the ridge markings were colored white, the valley markings were colored black, and both were presented against a medium gray background. We then blurred each of those images to smooth out the pattern of results with a Gaussian filter that had a radius of 24 pixels. Two of the observers participated in a second experimental session, which made it possible to measure their test-retest reliability. The average correlation between the smoothed response images in those sessions (excluding the background) was 0.73. It is important to note that this provides an upper bound on the possible correlations between the response images and any other stimulus measure. We also compared the response surfaces for each possible pair of observers, and the average correlation was 0.56. The difference between the test–retest correlations and the correlations among different observers confirms that there were some individual differences on this task as shown in Figure 9, but that there was also a large amount of agreement. Because the degrees of freedom for these correlations are all in excess of 580,000, the probability of a type I error is less than 10^−10^.

Additional analyses were performed to compare the patterns of observers’ judgments with the 3D structure of the depicted object and the luminance field for each image. This was achieved by averaging the smoothed response surfaces for all seven observers to create a composite response image. The structure of that image represents the overall pattern of results, in which bright regions correspond to the perceived locations of ridges and dark regions correspond to the perceived locations of valleys. If there were multiple interpretations about the location of a ridge or valley like the ones shown in Figure 9, all of those would be represented in the overall average.

**Figure 10.**
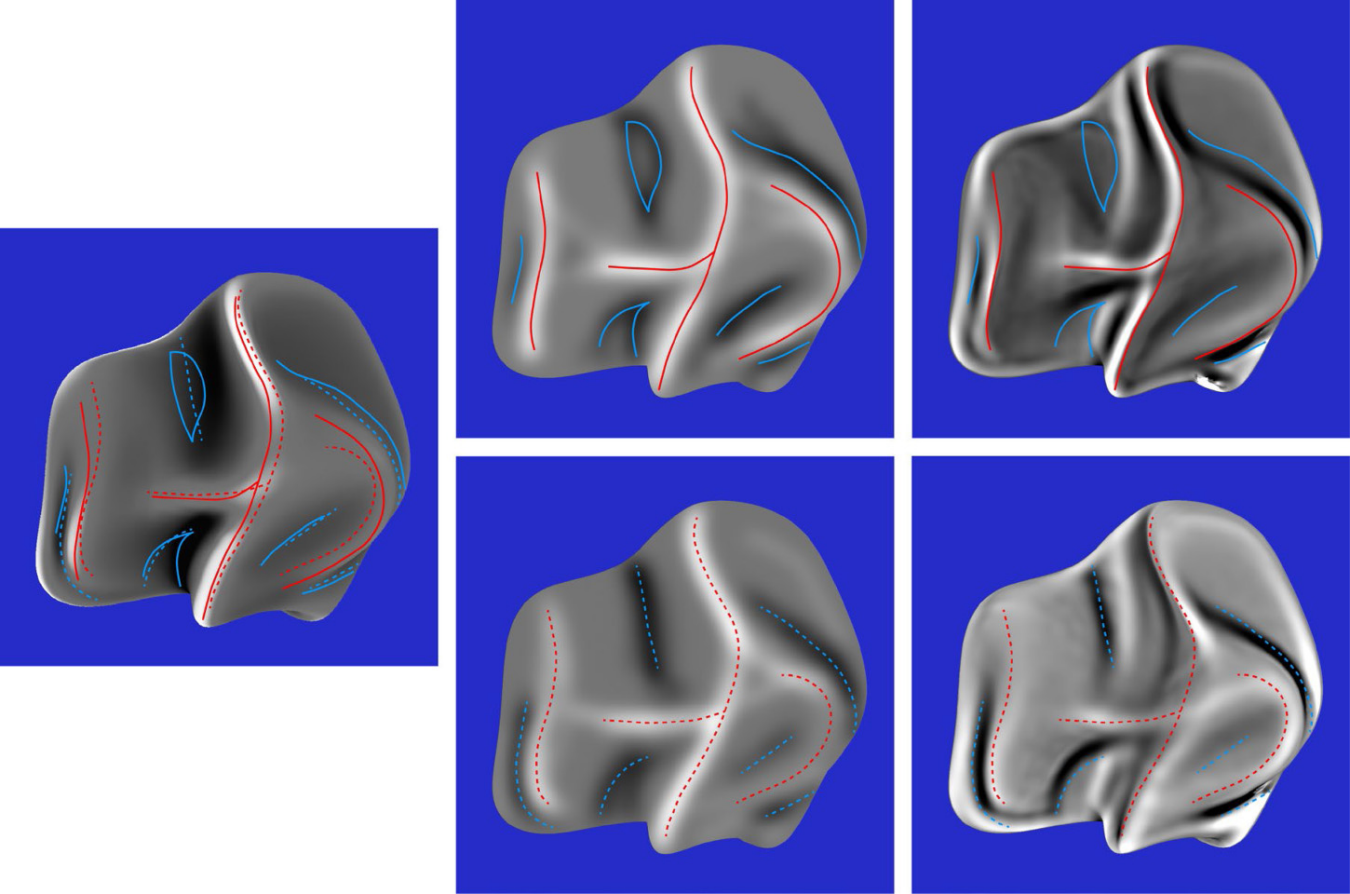
Left column: The mean curvature of one of the depicted objects. Middle column: The composite images from the observers’ judgments of ridges and valleys for the same object with left illumination (top) and right illumination (bottom). Right column: The mean curvatures of the luminance field for both possible patterns of illumination. The red and blue contours show the peaks and troughs of the composite response images.

**Figure 11.**
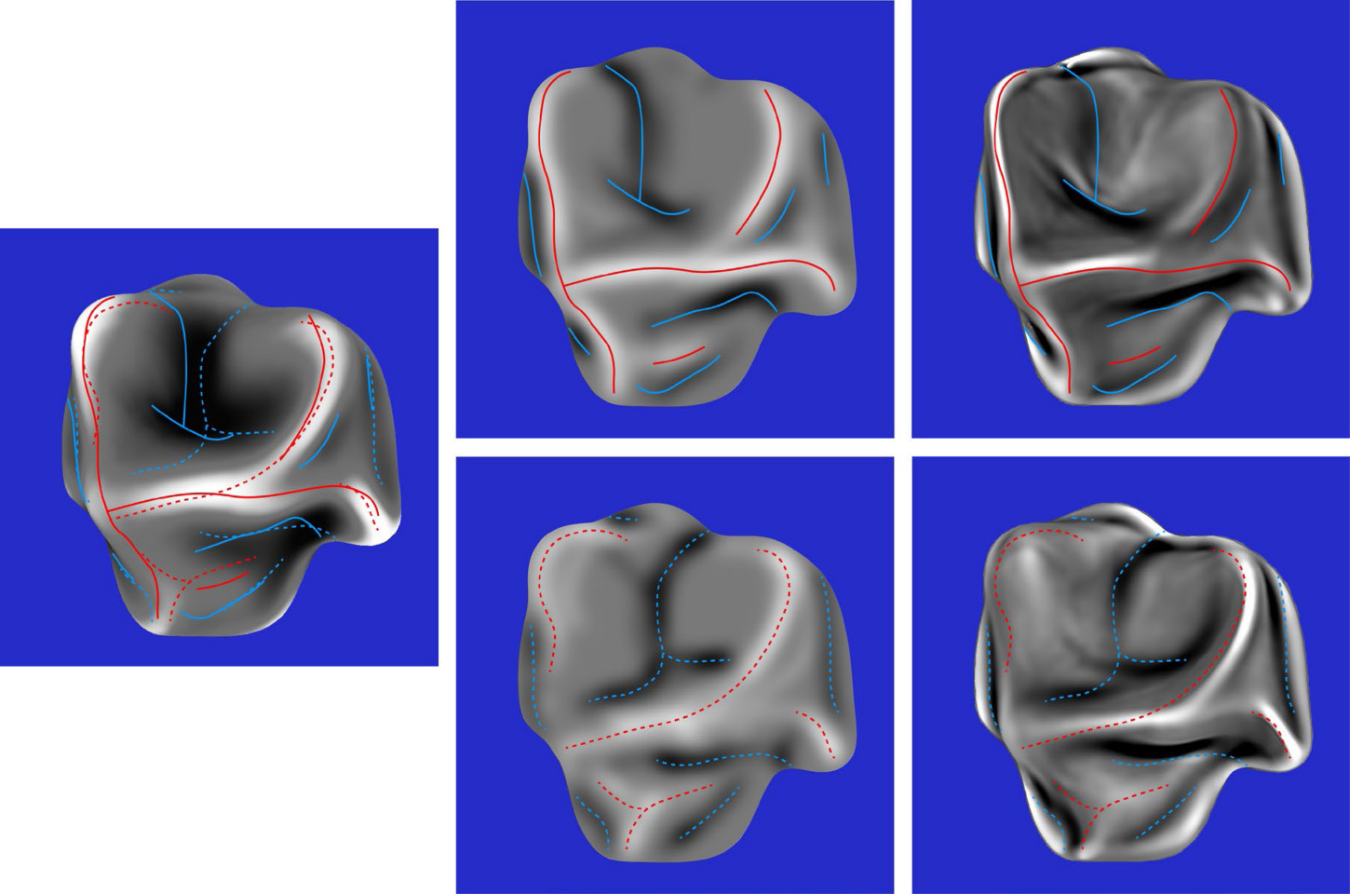
Left column: The mean curvature of one of the depicted objects. Middle column: The composite images from the observers’ judgments of ridges and valleys for the same object with left illumination (top) and right illumination (bottom). Right column: The mean curvatures of the luminance field for both possible patterns of illumination. The red and blue contours show the peaks and troughs of the composite response images.

**Figure 12.**
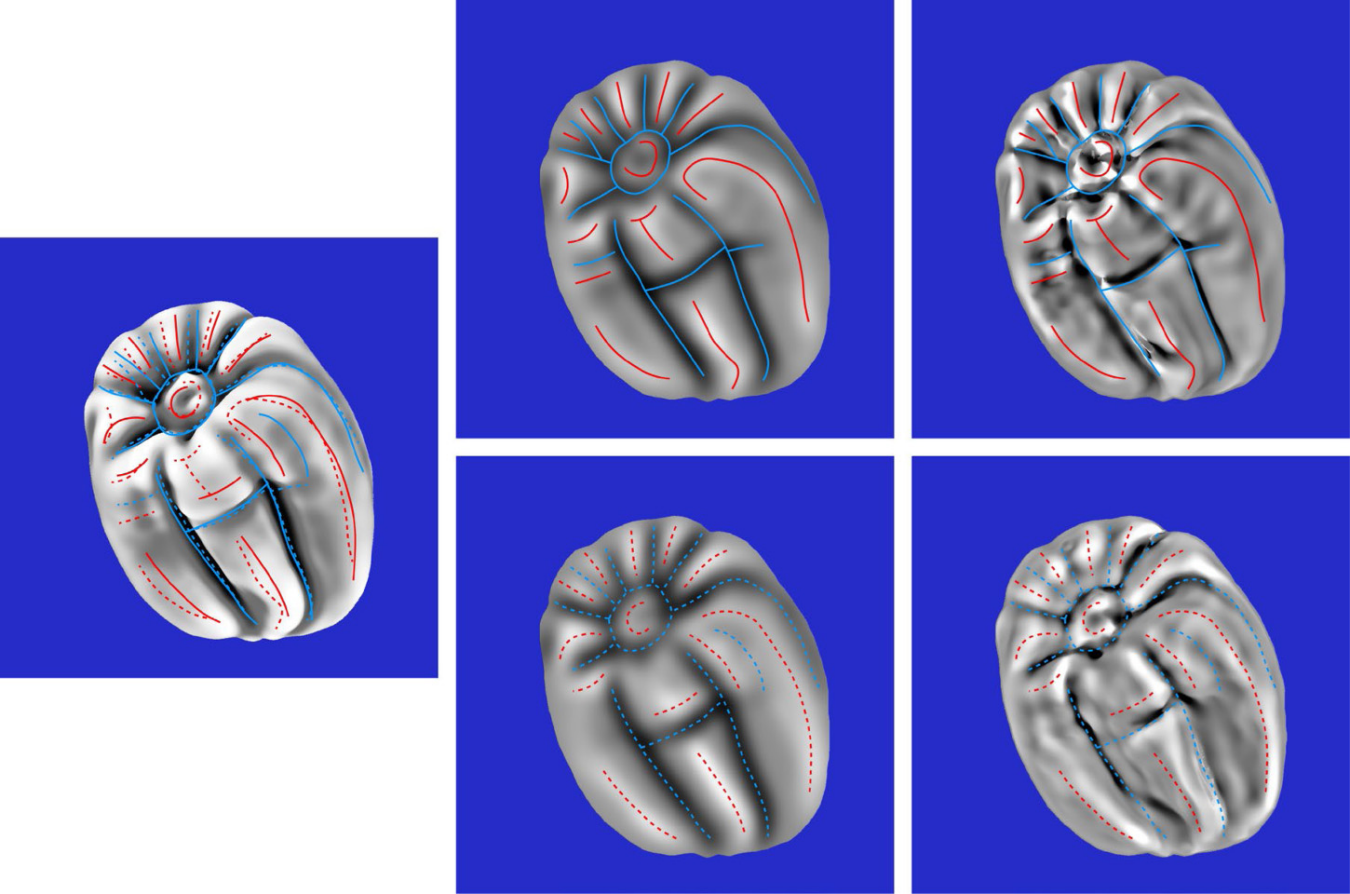
Left column: The mean curvature of one of the depicted objects. Middle column: The composite images from the observers’ judgments of ridges and valleys for the same object with left illumination (top) and right illumination (bottom). Right column: The mean curvatures of the luminance field for both possible patterns of illumination. The red and blue contours show the peaks and troughs of the composite response images.

The composite response images for all six stimuli are shown in the middle columns of Figures 10 to 12. The right columns of these figures show the mean curvatures of the luminance fields, where variations in gray scale are used to code the magnitude of curvature. Bright regions correspond to positive curvatures and dark regions correspond to negative curvatures. The patterns of red and blue contours superimposed on these curvature maps show the peaks and troughs of the composite response images. The left columns of these figures show the mean curvatures of the depicted objects. The solid and dashed contours show the pattern of responses for the left and right directions of illumination, respectively.

The most important thing to note in these figures is that the composite images of the observers’ responses in the middle columns look quite similar to the mean curvature maps of the depicted objects that are shown in the left columns, and that there is a high degree of constancy across the different patterns of illumination. The composite response images are less similar to the patterns of luminance curvature. Many of the judged ridges and valleys were closely aligned with luminance maxima and minima, which is consistent with the darker-is-deeper hypothesis of Langer & Zucker (1994). However, there were also many violations of that general rule. Some of the luminance curvature extrema were not marked by any of the observers (e.g., see Figure 10), even though they were clearly visible. Observers could apparently recognize that those features of the luminance field were not associated with the ridges and valleys on the depicted surface. There were also many instances where the observer's markings were shifted away from the luminance extrema in a systematic manner (see also Todd et al., 2023).

Additional quantitative analyses were performed to confirm these casual observations. The average correlation between the composite response image for each stimulus and the mean curvature of the depicted object was 0.71, which is close to the maximum possible value given the measurement error from the test-retest correlations. Similarly, the average correlation between the composite response image for each stimulus and the mean curvature of its luminance field was only 0.36. The obvious conclusion from these findings is that observers’ judgments must have been influenced by some other source of information in addition to, or instead of, the mean curvature of the luminance field. We also compared the composite response images for the same object with different patterns of illumination. The average correlation between the left and right illumination conditions was 0.78. which is quite remarkable given that the average correlations between the patterns of shading was −0.43. It is important to keep in mind that all of these correlations had at least 580,000 degrees of freedom. Thus, the probability of a type I error is less than 10^−10^.

## Discussion

Thus far we have presented a mathematical analysis to show that the number of qualitative features on a smoothly curved surface (e.g., ridges and valleys) is generally smaller than the number of corresponding features in its luminance field. We have also presented empirical evidence that observers are able to ignore the extraneous features within the luminance field so that the judged locations of ridges and valleys are in close correspondence with the depicted surface (see also Todd, Yu and Phillips, 2023). In the remaining portions of this paper, we will contrast the perceptions of human observers with the performance of computational models of shape from shading. We will consider an additional source of information that may facilitate shape constancy over different patterns of illumination. We will describe some possible physiological mechanisms for the measurement of luminance curvature, and we will discuss how the global organization of qualitative features may be related to the perception and production of line drawings.

### Computational Models of 3D Shape From Shading

Zhang et al. (1999) performed simulations of 19 different algorithms for computing shape from shading. All of these algorithms were based on an assumption that the direction and magnitude of the incident light is the same over all visible surface regions – what is sometimes referred to as homogeneous illumination. However, that assumption is almost never satisfied in natural vision because of the effects of cast shadows, multiple light sources and indirect illumination. When these models are tested with more realistic patterns of shading, they often produce erroneous features like ridges or valleys that are not physically present on the actual object. This is most likely due to the fact that changes in the sign of luminance curvature can be influenced by inhomogeneities in the pattern of illumination, and that those changes can be misinterpreted by computational models as changes in surface curvature.

Han, Zickler, and Nishino (2024) have recently developed a neural network model for computing shape from shading that uses a diffusion process to estimate local surface orientations within small patches of an image. Like most other computational models of shape from shading, the model assumes that a depicted surface has a Lambertian reflectance function, and that each patch is illuminated from a single dominant direction without shadows or indirect reflections. We wondered how this model might perform for the images shown in Figure 8 that violate its assumptions about the pattern of illumination. When we contacted the authors to get their thoughts on this issue, they graciously offered to run some simulations of their model with our stimuli, and to generate mean curvature maps by differentiating the estimated patterns of surface orientation.

**Figure 13.**
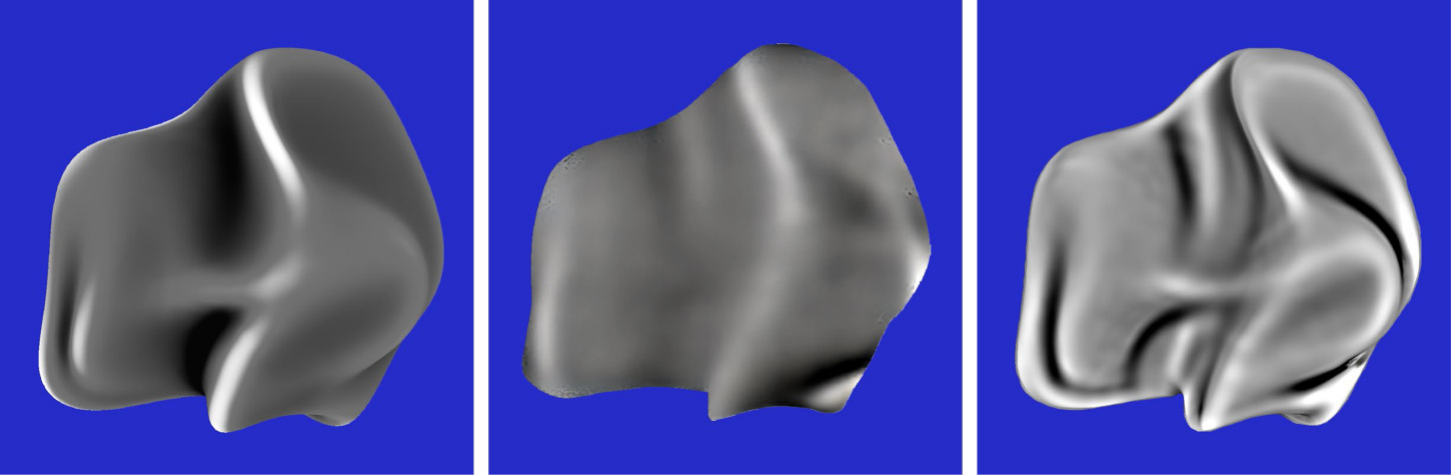
Left: The mean curvature of one of our stimulus objects. Middle: The estimated mean curvature of that object when it is illuminated primarily from the right. This image was provided by Xinran Han (with permission). Right: The mean curvature of the luminance field for the same pattern of illumination.

The results from one of those simulations are shown in the middle panel of Figure 13. It is flanked by a map of the mean surface curvature on the left and the mean luminance curvature on the right. Note that the model accurately captures the dominant vertical ridge through the center of the surface. However, it also detects a second ridge to the left of that, which is not present on the depicted surface, but is clearly visible in the pattern of luminance curvature. Another interesting aspect of the simulation is that it reverses the sign of curvature in the upper right quadrant.

It is important to keep in mind that this model was not designed to deal with multiple light sources, cast shadows, and indirect illumination, and it should not be surprising that it makes systematic errors when forced to analyze images that violate its underlying assumptions. These assumptions are widely adopted within the field of computer vision to make the computational analysis of shape from shading more tractable. However, it is difficult to rationalize those assumptions based on their ecological validity, because multiple light sources, cast shadows and indirect illumination are ubiquitous in the natural environment. The present experiments provide strong evidence that human perception is more robust to the complexities of real-world illumination, and that observers are somehow able to avoid the systematic errors produced by current computational models.

### Smooth Occlusion Contours

**Figure 14.**
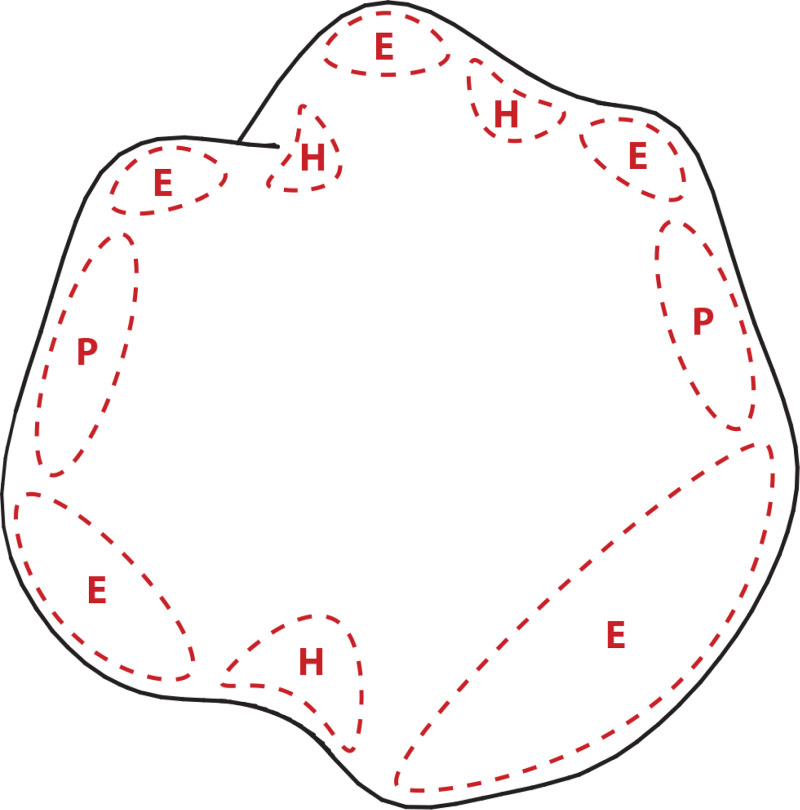
The rim of a smoothly curved object. The regions outlined by dashed lines in the interior are labeled as elliptic (E), hyperbolic (H) and parabolic (P) based on the curvature along the contour.

One possible source of information that could make that possible is provided by the smooth occlusion contour of an object, which is also referred to as the rim (Koenderink, 1984; Koenderink & van Doorn, 1982). The surface normals along smooth occlusion contours are all perpendicular to the line of sight. Moreover, the curvature perpendicular to the rim (on the attached side) is always convex. Thus, if a contour bends inward toward the object, then an attached surface region has a positive Gaussian curvature like a sphere (i.e., it is elliptic). If the contour bends outward away from the object, then an attached surface region has a negative Gaussian curvature like a saddle (i.e., it is hyperbolic), and if it is straight, then an attached surface region has zero Gaussian curvature like a cylinder (i.e., it is parabolic). Koenderink and van Doorn also showed that when the rim ends abruptly in an image, that can only occur in a hyperbolic region. Figure 14 shows the rim of a smoothly curved object presented in isolation. The red dashed curves in this figure show the signs of Gaussian curvature in adjacent regions along the contour. Note that the inner boundaries of these regions cannot be determined based solely on the rim. That requires additional sources of information, such as luminance curvature.

Smooth occlusion contours can also provide information about the direction of illumination (Ikeuchi & Horn; 1981; Todd & Reichel, 1989). For example, if the left side of an occlusion contour is brighter than the right side, it indicates that the primary illumination is from the left. Whenever that occurs, the luminance extrema will be shifted to the left of the peaks and troughs of the ridges and valleys. If observers are sensitive to that information, it could explain why the judged locations of the ridges and valleys were often systematically shifted relative to the curvature extrema of the luminance field. Todd et al., (2023) have shown that these shifts do not occur when an object is illuminated from a frontal direction.

### Possible Physiological Mechanisms

**Figure 15.**
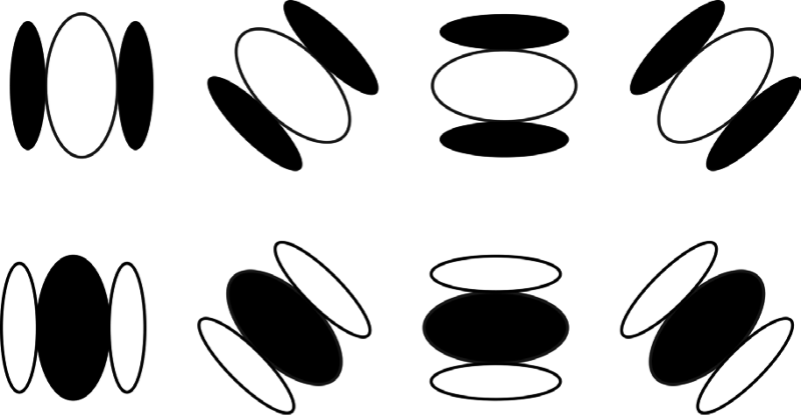
Receptive fields of luminance curvature detectors. Those in the top row respond best to bright regions that are flanked by darker ones. Those in the bottom row respond best to dark regions that are flanked by brighter ones.

An obvious question to consider in the context of the present discussion is whether it is physiologically plausible that the human brain uses differential geometry to analyze the patterns of light intensity that stimulate the retina. The answer to that question is unequivocally yes. In the late 1950's, David Hubel and Torsten Wiesel discovered that many cells in the primary visual cortex respond to oriented bars or gratings at a particular orientation. These cells have receptive fields that are remarkably similar to the Gabor filters used by engineers for signal processing. Figure 15 shows a set of these filters tuned to different orientations. The filters in the top row of the figure respond most vigorously when a region of high luminance is flanked by regions of lower luminance. The ones in the bottom row respond most vigorously when a region of dark luminance is flanked by regions of higher luminance.

Note that these cells can also be thought of as detectors of luminance curvature (Koenderink & van Doorn, 1990). The ones with receptive fields like those in the top row of Figure 15 respond best to positive luminance curvatures in a particular direction, and the ones with receptive fields like those in the bottom row respond best to negative luminance curvatures. Suppose that a large number of these cells with receptive fields in the same local neighborhood competed with one another, so that the ones that respond most vigorously suppress the outputs of all the others. The survivors of that competition would reveal the principal directions and magnitude of luminance curvature in each local region.

### Implications for Pictorial Art

**Figure 16.**
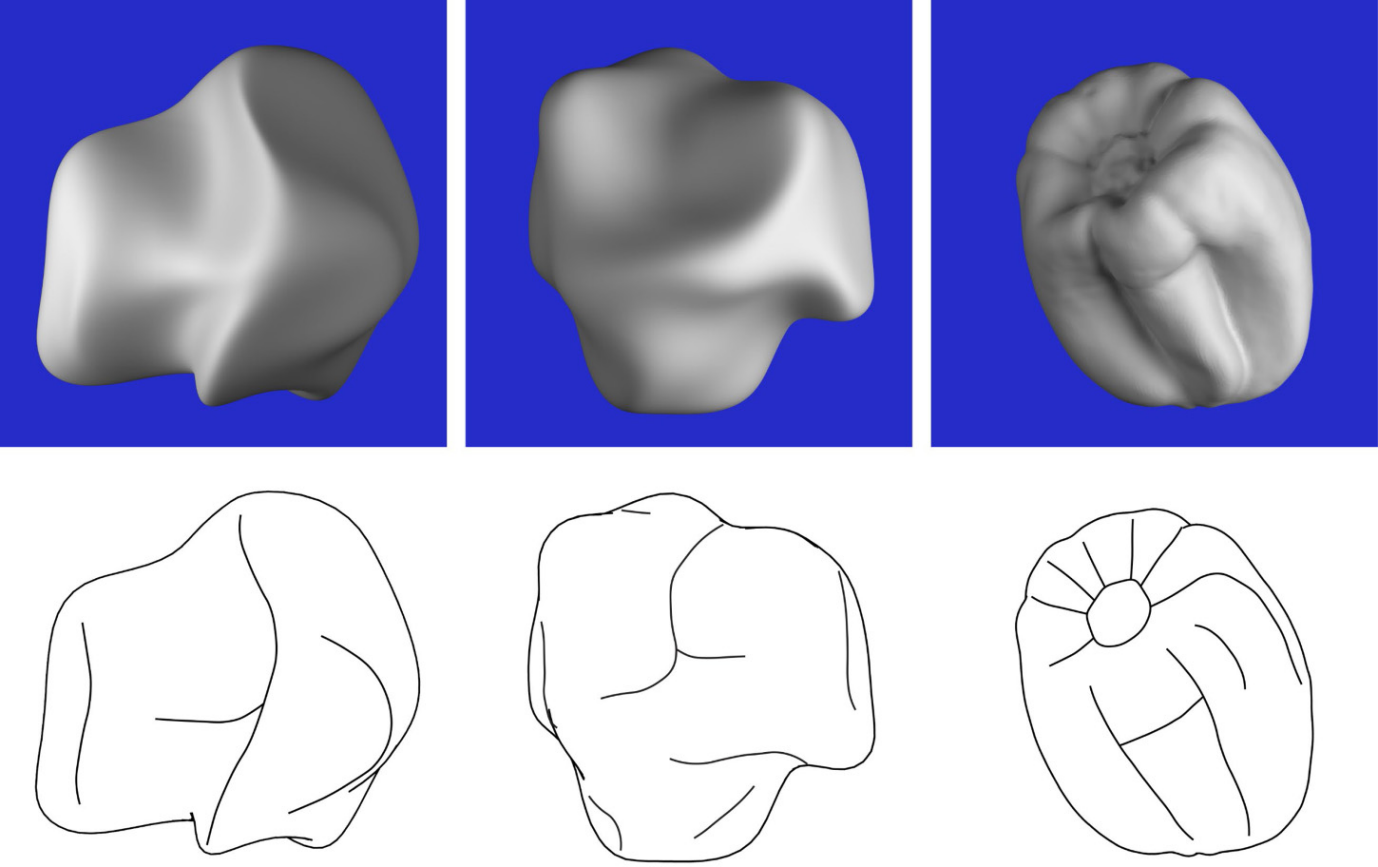
Top row: Three of the stimuli used in Experiment 2. Bottom row: Line drawings of these stimuli from the composite response images shown in Figures 10 and 12. The drawing on the left shows the judged ridges, whereas the other two drawings show the judged valleys.

We suggested in the introduction that the pattern of ridges and valleys on an object has a graph-like structure that can be used to depict surfaces in line drawings. To test that idea, we created line drawings from the composite response images shown in Figures 10 to 12. We quickly discovered that when the ridges and valleys were both presented in combination, the resulting drawings were far from convincing. Much better results can be achieved by only presenting the ridges (or valleys) in isolation. Figure 16 shows several examples. The most effective drawing for the object on the left is one that depicts the pattern of apparent ridges. However, for the other two objects, the most effective drawing depicts the pattern of apparent valleys.

There is an interesting line of research in computer graphics and human perception about where contours should be drawn to provide the most effective line drawings (e.g., Cole et al., 2008, 2009; DeCarlo et al., 2003; Hertzmann, 2020; Iverson & Zucker, 1995; Pearson & Robinson, 1985; Phillips et al., 2003). Almost all drawings of objects include smooth occlusion contours and the creases that occur where two surfaces intersect one another, but those are not sufficient for a convincing pictorial representation of smoothly curved surfaces. There are two basic proposals in the literature for addressing this problem. They both agree that contours should be derived from patterns of surface curvature, but they disagree about which aspects of differential structure should be highlighted.

**Figure 17.**
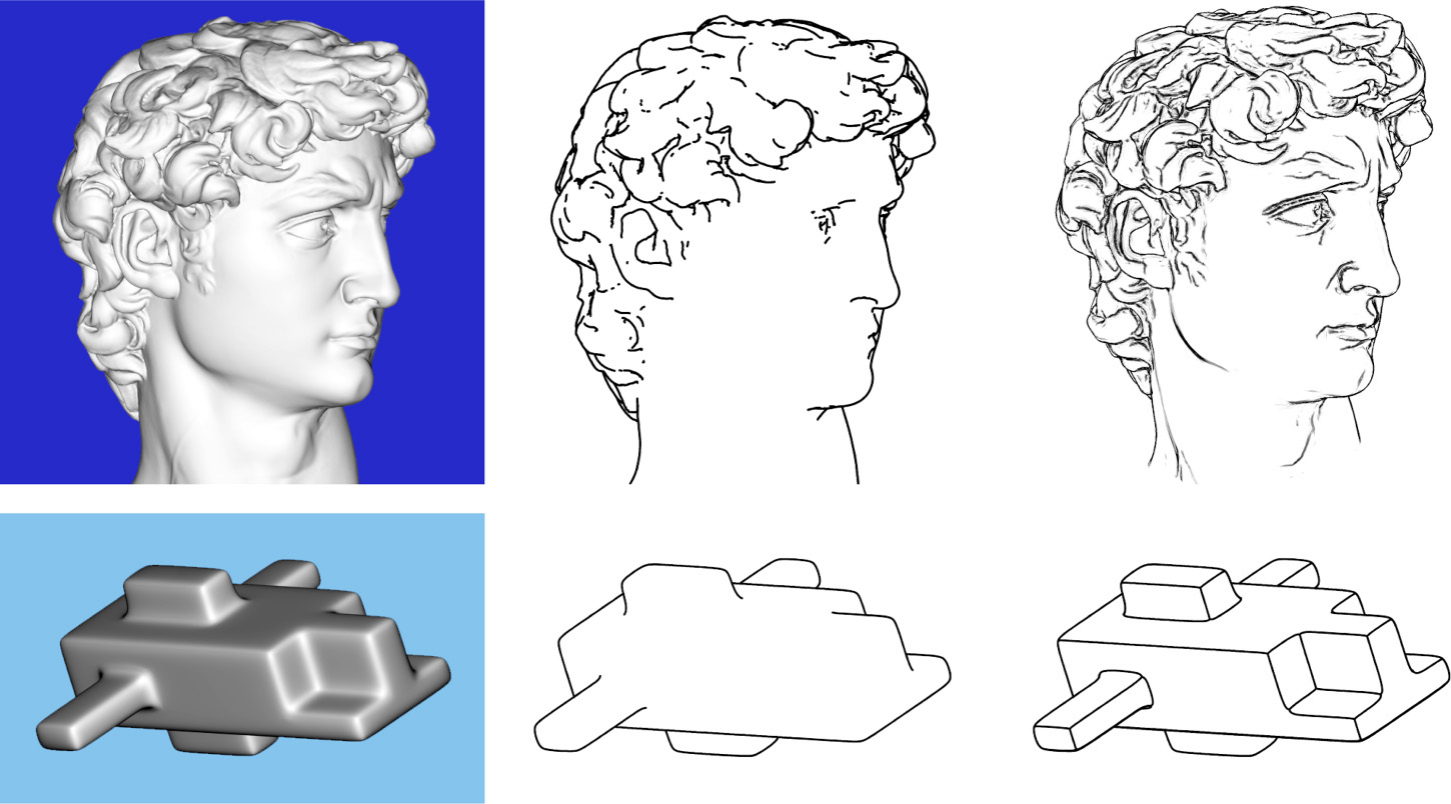
Top row: The left panel shows a shaded image of the bust of David created from a mesh provided by the Digital Michelangelo Project, Stanford University, with permission. The middle panel shows the smooth occlusion contours for this surface created with Finaltoon by Cebas. The right panel shows the occlusion and suggestive contours presented together. This image was provided by Doug DeCarlo, with permission, and it can be found at https://gfx.cs.princeton.edu/proj/sugcon/models/. Bottom row: The left panel shows a shaded image of a complex polyhedron with chamfered edges. The middle panel shows its occlusion contours presented in isolation, and the right panel adds curvature extremal contours.

One well-known approach was developed by DeCarlo et al. (2003). The traditional concept of a smooth occlusion contour (i.e., the rim) includes all points where the surface normal is orthogonal to the line of sight. DeCarlo et al. proposed a generalization of that concept to include all points where the surface depth gradient as defined by Equation 2 is a local maximum (see also Cole et al., 2008, 2009). The contours that result from this generalization are called suggestive contours. It is important to note that these contours correspond to parabolic lines on the depicted surface, because points with maximum local slant will always have a principal curvature of zero. The top row of Figure 17 shows a shaded image of the bust of David by Michelangelo, and two possible line drawings of it. The one in the middle panel shows only the smooth occlusion contours, whereas the one on the right also includes suggestive contours. The addition of suggestive contours clearly produces a more compelling impression of 3D shape.

Phillips et al. (2003) proposed a different technique for depicting smoothly curved surfaces with line drawings that involves generalizing the concept of a crease. This is traditionally defined as a discontinuity of surface orientation where two faces of an object are connected. Phillips et al. argued that this can be generalized to include regions where changes in surface orientation (i.e., curvature) are a local maximum, and that line drawings of smooth surfaces can be improved by plotting curvature extremal contours that connect points where one principal curvature (*K*_max_) is a local maximum, or the other principal curvature (*K*_min_) is a local minimum. This is implicit in line drawings of polyhedra. The creases have infinite curvature, and the faces have no curvature at all. Of course, this is impossible for real objects, but it is a reasonable approximation in many contexts. By generalizing the concept of a crease on polyhedral surfaces to include curvature extremal contours on more rounded surfaces, it is possible to enhance their pictorial depictions with line drawings. The bottom row of Figure 17 shows a shaded image of a complex polyhedron with chamfered edges and two line drawings of it. The middle panel shows the rim of this object presented in isolation. Note that it does not appear perceptually as a 3D surface. There are no suggestive contours on this object because the regions between the chamfered edges are uniformly flat. However, the addition of curvature extrema contours as shown in the right panel produces a much more compelling perception of 3D shape.

Although these approaches are useful for the design of non-photorealistic renderers, they are fundamentally inadequate to explain how human artists create line drawings. The problem with these analyses as psychological models is that they require a detailed representation of the ground truth in order to identify the relevant contours to draw in an image. However, when skilled artists create line drawings, they have no access to the ground truth. Their decisions about where lines should be placed must be determined entirely from sensory information.

One possibility might be to draw lines that correspond to features of the luminance field rather than those on the actual surface. DeCarlo et al. (2003) considered the case where a Lambertian surface is illuminated by a point light source located along the line of sight. Under those circumstances, local maxima of surface slant in the direction of the gradient will also be local negative extrema of luminance curvature. Hertzmann (2020) has pursued this idea further by arguing that observers interpret line drawings as if they were shaded images of Lambertian surfaces illuminated along the line of sight.

**Figure 18.**
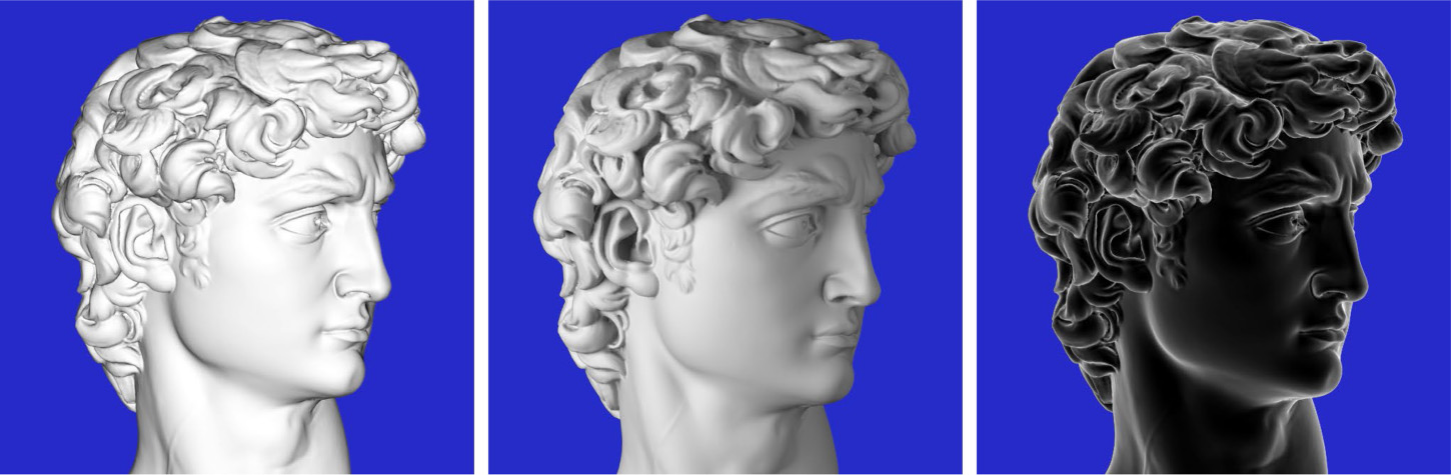
Three images of David. Left: The object is illuminated by a distant point light source along the line of sight. Middle: The object is illuminated by small area lights to the left and right. Right: A negative image of the one on the left.

Given that illumination along the line of sight is vanishingly rare in the natural environment, it is highly unlikely that the human visual system would adopt that as a prior for the perceptual interpretation of line drawings. Frontal illumination is degenerate in several important respects. It is the only pattern of lighting that does not produce cast shadows, and it also causes the frequency of light-to-dark changes in an image to double relative to more oblique illuminations (Langer & Bülthoff, 2000; Pentland, 1989; Todd, Egan, & Kallie 2015). Consider the two images of David in the left and middle panels of Figure 18. The one on the left is illuminated by a distant point light source along the line of sight, whereas the one in the middle is illuminated by a bright key light on the left and a dimmer frame light on the right. Note how the shading from frontal illumination appears less natural, and that the contour pattern of the hair appears more complex because of the frequency doubling. This can be revealed by paying close attention to the shading of the ear in both images. Pairs of negative luminance extrema from frontal illumination are merged into one for oblique illumination, giving it a less complex appearance. Although the negative luminance extrema in that case do not correspond to parabolic lines on the surface, they are still perceptually informative (see also Iverson & Zucker, 1995; Pearson & Robinson, 1985).

It is also reasonable to question whether there is anything special about negative luminance extrema for defining the structure of contour drawings. It is important to keep in mind that there is a general topological constraint that any two negative extrema must always be separated by a positive one, and vice versa. For images of densely packed ridges and valleys, like the ones in Figure 18, the negative and positive extrema will form very similar patterns. If the negative extrema are more sharply articulated than the positive ones, then they might be a better choice for defining the contours of line drawings, but that is not always the case. Consider, for example, the complex polyhedron depicted in the bottom row of Figure 17. The contours on that object are defined by positive luminance extrema. Another example is shown in the right panel of Figure 18, in which the shading is inverted relative to the left panel. This is similar to the patterns of reflectance that occur on surfaces composed of black velvet cloth (Koenderink & Pont, 2003). The positive luminance extrema are more sharply articulated than the negative ones, yet it still provides a dramatic depiction that is clearly perceived as the bust of David. Still another interesting example is provided by the images of bumps in Figure 6. Note that the prominent valleys on these surfaces are defined by positive luminance extrema on one side of the bump and by negative luminance extrema on the opposite side.

## Conclusions

The research described in the present article has examined the perception of ridges and valleys by human observers under variable conditions of illumination. We began by discussing how differential geometry can be used to mathematically define those features on 3D surfaces, and how the same analysis can also be used to define the qualitative structure of the luminance field. The most important take home message from that discussion is that the number of ridges and valleys on a surface is almost always smaller than the number of comparable structures within its luminance field, and that this is the primary reason why existing computational models of 3D shape-from-shading produce extraneous ridges and valleys that are not present on the actual depicted object (e.g., see Figure 13).

We then considered whether human observers make similar errors when judging the 3D shapes of objects defined by patterns of shading. Previous methods for measuring the apparent shapes of surfaces have used judgments of relative depth or orientation within small local neighborhoods (e.g., Koenderink, van Doorn & Kappers, 1992, 1995; Todd & Reichel, 1989). Although it is possible to reconstruct a global 3D surface from such measures, the resolution is too coarse to pinpoint the locations of apparent ridges and valleys. In an effort to overcome that difficulty, we developed some alternative procedures in which observers marked the apparent boundaries of bumps, or the positions of ridges and valleys, without having to infer those structures from lower order measures. The results revealed that observers can accurately identify the locations of these features, and that there is a high degree of constancy across different patterns of illumination.

The relationship is much weaker between the perceived locations of ridges and valleys and the extrema of curvature within the luminance field. Computational models of shape from shading almost always assume that changes in luminance are solely determined by variations of surface orientation. However, this conveniently ignores the fact that similar changes in luminance can also be influenced by local variations in illumination caused by multiple light sources, cast shadows, or indirect reflections. Human observers can somehow distinguish those two components of luminance variation, but the visual information that makes that possible has yet to be determined.

An adequate theoretical understanding of the perception of 3D shape from shading will ultimately require a systematic analysis of the luminance field and how it is related to the 3D structure of a visible surface. Although a few promising first steps toward that goal have been described in the literature (e.g., Han & Zickler, 2024; Koenderink & van Doorn, 1980; Kunsberg & Zucker, 2018, 2021), they are all limited by restrictive assumptions about Lambertian reflectance and homogeneous illumination. It makes little sense to ground a theory of human perception on a set of assumptions that are almost never satisfied in the natural environment. This is analogous to the old joke about a group of economists who are trapped in a deep pit and plan their escape by assuming they have a ladder, or the one about a drunk who loses his keys at night under some bushes, but searches for them under a nearby streetlight because it is easier to see there. The standard assumptions for computing shape from shading may have made sense 40 years ago when the field was still in its infancy, but it is clearly time to broaden the scope of that research.

## Appendix

### The Computation of Curvature

A standard technique for computing the curvature of a scalar field is to fit a second order quadric surface patch at densely sampled locations using a least squares procedure. The patch is defined by the following equation: *Z* *=* *aX^2^* *+* *bY^2^* *+* *cXY* *+* *dX* *+* *eY*, where *X* and *Y* are the horizontal and vertical coordinates of a given location and *Z* is the height of a surface or the intensity of an image at that location. From the coefficients of the best-fitting patch, an analytic solution can be obtained for all aspects of curvature, including mean curvature, Gaussian curvature and the principal directions of curvature.

The curvature maps in the present paper were obtained using an open-source software package called MeshLab developed by the Institute of Information Science and Technologies of the Italian National Research Council. This software is designed for the processing of 3D surface meshes, so it is naturally suited for the stimulus objects used in our experiments. However, it is not designed for the processing of images. To circumvent that problem, we transformed each stimulus image into a mesh using a standard 3D modeling technique called displacement mapping. Points on a plane are displaced orthogonally at each location based on the intensity of a corresponding location within a shaded image. This effectively transforms a shaded image into a 3D surface mesh.

MeshLab provides tools for computing several different measures of curvature including κ_max_, κ_min_, mean curvature, and Gaussian curvature. These are output as color maps like the ones shown in Figure 6. For other figures with superimposed color lines, it was more effective to represent curvature using grayscale rather than color. This was achieved by transforming the red, green, and blue values (R, G, B) to hue, saturation and brightness (H, S, B), and creating new images from the brightness values.

Our original insight when we began this project is that κ_max_ would be the most appropriate measure for characterizing curvature extrema. However, there is a subtlety in how that measure is defined that we did not fully appreciate. Consider the two principal curvatures (κ_1_, κ_2_) at any given surface location. By convention, when one or both of those curvatures are negative, the one that is most positive is assigned to be κ_max_, as opposed to the one that is most curved. Because of that convention, maps of κ_max_ can have little or no variation of shading in regions of negative curvature. We therefore adopted mean curvature for the maps presented in this paper to avoid that problem. We suspect that an alternative measure might be more appropriate in this context that is defined as the principal component that is most curved as opposed to the one that is most positive.
